# Plant-Derived Vesicle-like Nanoparticles for Cancer Therapy: From Drug Delivery to Combined Immunotherapy

**DOI:** 10.3390/antiox15030311

**Published:** 2026-02-28

**Authors:** Jinying Zhang, Yuan Zuo, Bo Sun, Xinxin Wang, Shuo Tian, Mingsan Miao

**Affiliations:** 1 College of Pharmacy, Henan University of Chinese Medicine, Zhengzhou 450046, China; 2 Collaborative Innovation Center for Respiratory Disease Diagnosis and Treatment & Chinese Medicine Development of Henan Province, Zhengzhou 450046, China; 3 The Engineering and Technology Center for Chinese Medicine Development of Henan Province, Zhengzhou 450046, China

**Keywords:** plant-derived vesicle-like nanoparticles, engineering strategy, drug delivery, cancer immunotherapy

## Abstract

Plant-derived vesicle-like nanoparticles (PDVLNs) are a unique class of natural nanomaterials secreted by plant cells. Endowed with intrinsic biocompatibility, minimal immunogenicity, and a molecular cargo rich in lipids, proteins, nucleic acids, and unique metabolites, PDVLNs exhibit significant potential in cancer treatment. With the development of diverse engineering strategies and loading methods, PDVLNs have been well established as an ideal drug delivery platform for transporting a broad spectrum of anti-cancer agents, including nucleic acids, proteins, and conventional chemotherapeutics. Notably, accumulating evidence highlights PDVLNs as a novel nanoplatform for combined cancer immunotherapy. This review systematically summarizes the biogenesis, isolation methods, compositions, and anti-tumor activity and mechanisms of PDVLNs, emphasizing their synergistic applications with cancer immunotherapies. Finally, it also discusses the challenges related to production optimization, delivery efficiency, safety issues, and clinical translation facing current research. In the future, as mechanistic insights deepen and nanotechnology advances, PDVLNs are poised to become next-generation immunomodulatory nanoplatforms for enhanced cancer immunotherapy.

## 1. Introduction

Cancer remains the leading cause of mortality worldwide, posing an unprecedented burden on global healthcare systems [1,2]. Conventional treatment modalities such as chemotherapy, radiotherapy, targeted therapy, and immunotherapy are frequently limited by several challenges, including severe off-target toxicity, multidrug resistance, and poor prognosis [3,4]. The advent of nanomedicines, including mammalian-derived extracellular vesicles (MDVs) and synthetic nanocarriers (liposomes, polymers, and inorganic nanoparticles), presents a promising avenue for cancer treatment [5,6]. These nanocarriers are engineered to deliver therapeutic nucleic acids, chemotherapeutic agents, and immunotherapeutic agents to the tumor microenvironment (TME) [7]. By enabling precise targeting of tumor tissues, cells, and even subcellular organelles, they enhance therapeutic efficacy while minimizing systemic toxicity, thereby aligning with the core principles of precision medicine. However, their clinical application of are frequently plagued by issues such as immunogenicity, high cost, and complex manufacturing processes [8]. Furthermore, stringent regulatory review frameworks have significantly impeded the clinical translation of most candidate nanoparticles, posing substantial challenges to their approval process [9].

In recent years, advances in nanotechnology have increasingly directed scientific interest toward plant-derived vesicle-like nanoparticles (PDVLNs), a class of membrane-bound nanostructures secreted by plant cells that possess lipid bilayer architectures [10]. As a group of extracellular vesicles (EVs), natural vesicle-like nanoparticles (VLNs) typically exhibit diameters between 50 and 500 nm and efficiently encapsulate diverse bioactive lipids, proteins, RNAs, and other functional molecules, which contribute to their cross-kingdom communication within plants and across species [11,12]. In contrast to synthetic nanoparticles, PDVLNs offer advantages such as low immunogenicity, high biocompatibility, broad availability, and feasibility for large-scale isolation, rendering them a promising nanoplatform for natural therapeutics and delivery carriers [13,14].

Importantly, an increasing number of studies demonstrate that PDVLNs have garnered significant attention in cancer treatment, such as lung carcinoma, glioblastoma, colon cancer, breast cancer, and hepatocellular carcinoma [15,16,17]. Certain PDVLNs or the bioactive compounds within them can suppress tumor progression through multiple mechanisms. As natural anti-tumor agents, they can induce regulated cell death (RCD), arrest cell cycle, regulate gut microbiota, and even reverse immunosuppressive TME [18]. In addition, innate targeting capability and biocompatibility render them optimal drug carriers for small-molecule drugs, proteins, and nucleic acids, thereby potentially improving the efficiency of site-specific delivery [10]. Apart from their natural applications, rational engineering strategies have endowed them with additional functions, such as enhanced tumor targeting ability and tissue penetration ability [19].

Despite the transformative advances of cancer immunotherapy in revolutionizing cancer treatment paradigms, several intractable limitations continue to severely impede their clinical efficacy, such as tumor immune escape, low response rates, and immune-related adverse events [20]. In this context, PDVLNs, as a natural drug delivery vector, can effectively overcome these limitations when integrated with cancer immunotherapy, demonstrating a unique dual functionality. They not only function as intrinsic immunomodulators capable of reprogramming the immunosuppressive TME, but also serve as efficient co-delivery platforms that synergize with diverse immunotherapeutic agents, thereby enhancing overall therapeutic efficacy [19].

Although some reviews have summarized the anti-cancer potential of PDVLNs, there remains a relative paucity of reviews on their role in combinational cancer immunotherapy. Here, the paper offers a retrospective overview of the fundamental properties of PDVLNs, covering biogenesis, isolation methods, components, as well as their anti-tumor activities and mechanisms. We will concentrate on the recent advances in engineering PDVLNs as anti-tumor drug carriers and their application in combinational cancer immunotherapy. Finally, we will discuss the current challenges and prospects associated with their standardization, clinical transformation, and combinational cancer immunotherapy.

## 2. Basic Properties of PDVLNs

### 2.1. Biogenesis of PDVLNs

**Figure 1:**
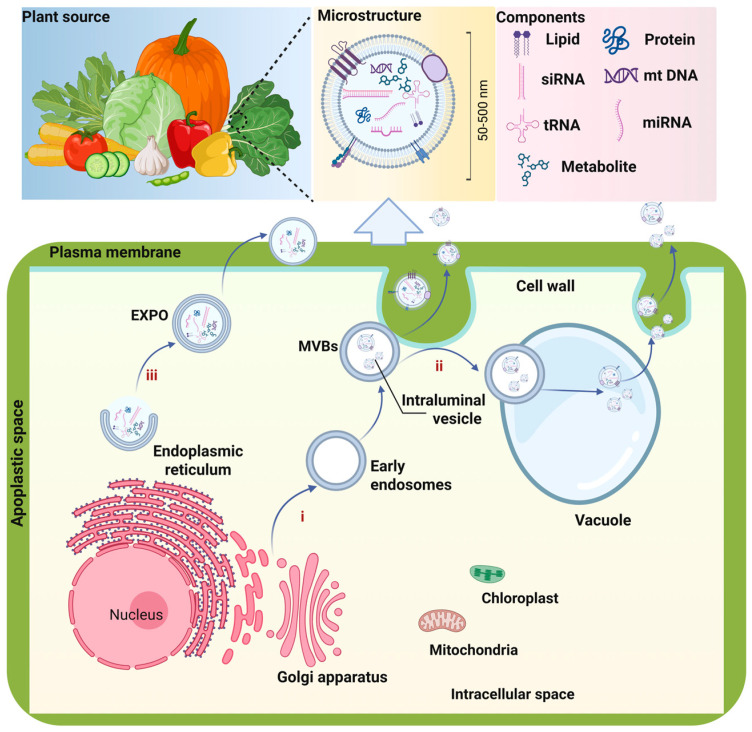
The biogenesis pathways and components of PDVLNs. The biogenesis pathways of PDVLNs in plant cells: (i) the multivesicular bodies (MVBs) pathway; (ii) the vacuolar pathway; (iii) the exocyst-positive organelle (EXPO) pathway. EXPO, exocyst-positive organelle, MVBs, multivesicular bodies.

PDVLNs have emerged as a prominent area of nanomedicine research in recent years, with their biogenesis garnering significant attention. Research has shown that the biogenesis pathways of PDVLNs include three types: the multivesicular bodies (MVBs) pathway, exocyst-positive organelle (EXPO) pathway, and vacuolar pathway [21], as shown in Figure 1. Among them, (i) the MVBs pathway is considered the primary biogenic pathway of PDVLNs. In 1967, Cui et al. [22] first observed MVBs in carrot cells using a transmission electron microscope (TEM). MVBs function as late endosomes (LEs) that encapsulate intraluminal vesicles (ILVs), which are formed through the limiting membrane invagination and budding into the lumen. MVBs fuse with the plasma membrane (PM) and lead to the release of their ILVs into the extracellular environment, which is considered the main route mediating vesicle secretion. Additionally, discovered that *Arabidopsis*-derived VLNs were also released through MVBs [23]. (ii) Another pathway involved in PDVLNs secretion, known as the EXPO pathway, was identified by Wang et al. EXPO, as a spherical double-membrane organelle, was found to be fused with PM and release a single membrane vesicle into the cell wall. The EXPO pathway is an unconventional secretion pathway unique to plants [24]. *Arabidopsis* and tobacco-derived VLNs are produced through this pathway. (iii) In addition to the spontaneous paths of MVBs and EXPO, vacuoles can be considered a passive mode of generation. Vacuoles, as an important single-membrane organelle, play an important role in plant defense owing to their hydrolytic enzyme content [25]. When plants are attacked by pathogens, vacuoles are released into the extracellular environment by fusion with the PM and release hydrolases and defense proteins into the extracellular space [26]. A recent report indicated that the formation of central vacuoles in plant cells begins with the transformation of MVBs into smaller vacuoles that subsequently unite to generate a more prominent vacuole [27]. In addition, autophagosomes, arising from apoptotic cells, may fuse with MVBs, followed by fusion with the plasma membrane to release EVs, have also been suggested as one of the possible pathways [28]. However, the autophagosome pathway still lacks sufficient evidence in plant cells. Diverse biogenesis pathways of PDVLN contribute to the heterogeneity among PDVLN subtypes. Therefore, more investigations are needed to reveal these biogenetic mechanisms.

### 2.2. The Preparation of PDVLNs

#### 2.2.1. Plant Sample Pretreatment

**Figure 2:**
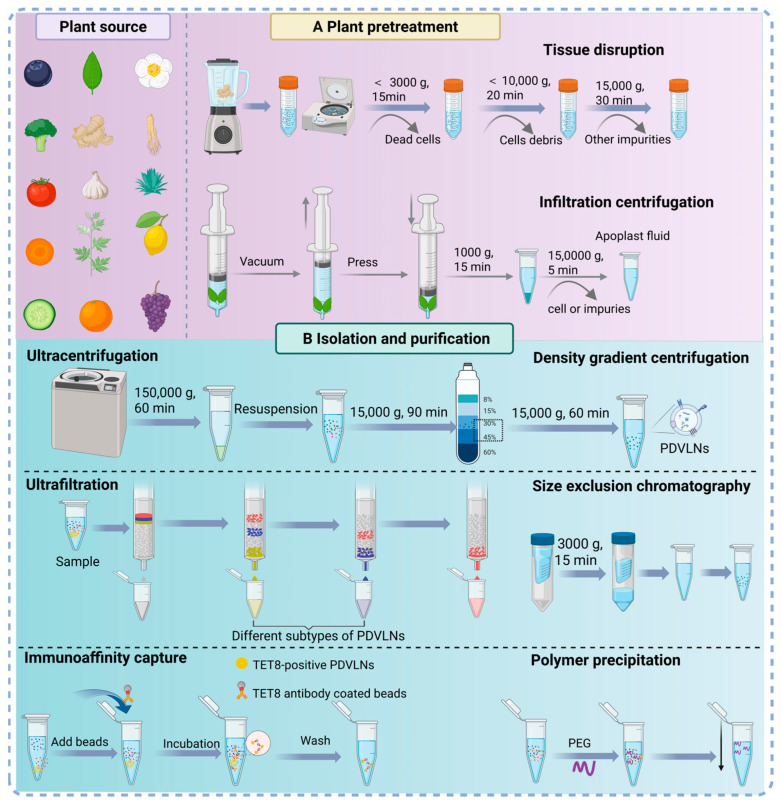
The tissue pretreatment, isolation, and purification strategies for PDVLNs. (**A**) Plant pretreatment. Tissue disruption to obtain juice and tissue infiltration method to obtain the apoplastic washing fluid. (**B**) Isolation and purification methods. PEG, polyethylene glycol.

Before extracting PDVLNs, the proper pre-treatment of plant tissues from edible or medicinal plants is usually conducted, which mainly involves plant tissue disruption in a blender and mixture with phosphate buffer saline (PBS) for obtaining plant juice [29]. The fresh plant juice is subjected to multiple steps of low-speed centrifugation to eliminate excess cell debris and large organelles (Figure 2A). Although tissue disruption is adopted by most researchers due to its high yield of PDVLNs, the potential cell disruption may increase the risk of contamination by other organelles and impurity proteins, thus decreasing the purity of PDVLNs. For example, Liu et al. [30] performed a proteome study of leaf VLNs and identified several proteins known to normally exist in the cytosol, nucleus, and intracellular organelles, which suggested that the co-isolation of non-vesicle material with other impurities was caused by cell disruption. Another preprocessing strategy, the tissue infiltration centrifugation method, is worth considering, which is originally utilized to explore plant metabolism from apoplast fluid of plant cells [29]. The method has been utilized to purify VLNs from apoplast fluids of sunflowers and *Arabidopsis* [31,32]. In general, intact plant tissues are immersed in an infiltration buffer, and vacuum equipment is used to obtain apoplast fluid, preventing mechanical damage to the plant cells and ensuring higher purity of PDVLNs [33]. However, the plant material amenable to this method is limited to plant leaves due to the action principle. Other plant tissues (e.g., flowers, roots, and stems) have rarely been reported. To ensure high PDVLNs yield and purity, multiple factors need to be taken into account, such as the type of plant material, centrifugation parameters, and juicing time.

#### 2.2.2. Isolation Methods of PDVLNs

Efficient and standard extraction and separation methods are prerequisites for the exploitation and application of PDVLNs. Different isolation and purification methods have distinct advantages in acquiring PDVLNs (Table 1). Similarly to MDVs, various extraction and purification techniques have been applied to obtain PDVLNs with high restoration, high purity, and low cost, which include ultracentrifugation (UC), density gradient centrifugation (DGC), ultrafiltration (UF), size exclusion chromatography (SEC), immunoaffinity capture, and polymer precipitation (Figure 2B). Among them, UC is the most widely used and is regarded as the “gold standard” for exosome preparation due to its cost-effectiveness, high yield, and the removal of extensive plant tissues and cellular debris [34]. However, continuous centrifugation may require an extended duration and could potentially result in the disruption of vesicle structure and the aggregation and precipitation of various EV subtypes [35]. Therefore, DGC is very necessary to further distinguish different EV subclasses or vesicles with similar size [36]. After UC, PDVLNs with different features are separated and distributed in the intermediate layer of a sucrose solution with concentrations between 30% and 45% [37]. The entire procedure is conducted at 4 °C and, while effective, remains labor-intensive and time-consuming, and is more suitable for small-scale production and functional research [38].

Other techniques, such as UF and SEC, are commonly employed for isolating PDVLNs as well. UF separates samples by leveraging molecular weight and size of PDVLNs using a nano-porous membrane with different cut-off values [21], although it carries the risk of degrading PDVLNs during the process. SEC does not exert excessive external force on the solvent, allowing PDVLNs to maintain their intact structure and biological activity [39], but it necessitates specialized equipment and can yield lower amounts of isolated material at one time [40,41]. Combining SEC with UF can enhance both the purity and functionality of PDVLNs [42]. Immunoaffinity capture mainly relies on the combination of specific covalent or high-affinity magnetic beads with the specific marker on the surface of PDVLNs, achieving up to 98% purity [43]. Polymer precipitation uses polyethylene glycol as a co-precipitant to reduce solubility, thereby facilitating the aggregation and subsequent precipitation of PDVLNs [44]. While the method offers distinct advantages, including operational simplicity, rapid processing, and suitability for large-scale production, it often leads to lower purity due to the co-isolation of non-target proteins and low specificity [45].

**Table 1:** The characteristics of isolation and purification methods for PDVLNs.

Preparation Techniques	Principles	Advantages	Disadvantages	Refs
Differential ultracentrifugation	Difference in density	Simple operation; low cost; high sample processing capacity; excellent yield	(i) Lengthy duration (ii) Low purity (iii) Special equipment required	[46]
Density gradient centrifugation	Precipitation velocity and density	High purity, minimal contamination; separation of a subpopulation of exosomes	(i) Time and labor-consuming (ii) High equipment requirement (iii) Complicated operation steps	[47]
Ultrafiltration	Particle size	High efficiency; great portability	(i) Highly complex procedure; (ii) Destruction of PDVLN structure (iii) Possibility of clogging	[48]
Size-exclusion chromatography	Particle size and molecular weight	High purification; suitable for separation of large diluted samples; great reproducibility	(i) Lengthy duration (ii) Lack of specificity (iii) Complex equipment required	[39]
Immunoaffinity capture	Specific binding of PDVLNs to affinity magnetic beads	High purity (≥99%) and selectivity; convenient operation and great enrichment	(i) Longer processing time; higher cost (ii) Low yield (iii) High requirements for reagents	[49]
Polymer precipitation	Reduce the solubility of PDVLNs to cause precipitation	Simple operation; shorter processing time; preservation of PDVLN integrity	(i) Retention and contamination of the polymer	[50]

### 2.3. Key Active Components of PDVLNs

#### 2.3.1. Lipids

An increasing number of lipidomics results have identified that the major lipid components of PDVLNs were composed of phosphatidic acid (PA), phosphatidylcholine (PC), phosphatidylethanolamine (PE), monogalactosyldiacylglycerol (MGDG), triglyceride (TG), and ceramide (Cer) [51]. These lipids constitute the essential components of the bilayer membranes of PDVLNs and play crucial roles in promoting nanovesicle formation, enhancing membrane fusion, and mediating cellular signal transduction [52,53]. For example, the application of exogenous glycosyl-inositol-phospho-ceramides onto leaf surfaces directly promotes PDVLN biogenesis and release, providing valuable insights into the biogenesis of PDVLNs. In addition, Ginger-derived VLNs (GVLNs) rich in PA were preferentially internalized by *Lactobacillaceae* in a lipid-dependent manner and regulated various targeted genes in *Lactobacillus rhamnosus* [16]. These lipids usually exhibit specific biological properties in inflammation-related diseases and cancer. In another study, the research team showed that PA (34:2) in GVLNs could mediate their absorption by the periodontal pathogen *Porphyromonas gingivalis*, and interacted with hemin-binding protein 35 (HBP35) on the surface to alleviate periodontitis [54].

In addition to anti-inflammatory effects, the lipids in PDVLNs have potential anti-tumor applications. Previous research showed that MGDG present in *Morus nigra* L. leaves-derived VLNs (MLVLNs) was identified as a signal molecule. MGDG exhibits specific affinity for the asialoglycoprotein receptor, a receptor overexpressed on the surface of liver tumor cells, thereby facilitating the liver tumor-targeted delivery of bioactive ingredients [55]. Moreover, Cao et al. [56] revealed that digalactosyl monoacylglycerol, PE, and Cer are involved in inducing tumor-associated macrophages to switch from the M2 phenotype to the M1 phenotype via a TLR4-MyD88-dependent pathway.

#### 2.3.2. Proteins

Evidence has demonstrated that proteins of PDVLNs are usually classified into three categories, including peripheral membrane proteins, transmembrane proteins, and intracellular proteins. They play a crucial role in mediating the physiological processes and EV secretion of plants. For instance, Ou et al. [57] identified 1843 proteins involved in the secretion of PDVLNs and signal processes in *Catharanthus roseus* (L.) Don leaves, and six marker proteins were suggested as potential marker proteins. Aquaporin, as a transmembrane protein that helps maintain intracellular osmotic pressure homeostasis, was enriched in Citrus clementina fruit juice-derived nanovesicles [58]. Similarly, the protein was present in broccoli-derived VLNs and contributed to membrane stability and cellular uptake [59]. After elimination of surface proteins by trypsin digestion, garlic-derived VLNs showed a lower level of cellular uptake compared with the undigested group. Specifically, mannose-specific binding protein, II lectin, located on the membrane surface of garlic-derived VLNs, interacted with CD98 on the cell membrane surface, thereby promoting their uptake efficiency [60].

Proteins in PDVLNs function as the bioactive substances to show potential anti-tumor properties. Recently, Cui et al. [22] reported the three proteins shared by *Arabidopsis thaliana*, olive, tobacco, and sunflower VLNs, including heat shock protein (HSP 70), S-adenosine homocystase, and glyceraldehyde 3-phosphate dehydrogenase. Among them, HSP 70 was found to be associated with tumor invasion and treatment resistance in human cancers [61]. Furthermore, proteins from GDNPs may be associated with the induction of the polarization of M2 macrophages into M1 macrophages, contribute to the modulation of the immunological microenvironment, and inhibit the progression of melanoma [56].

#### 2.3.3. Nucleic Acids

Studies have shown that PDVLNs are enriched with nucleic acids, including DNAs and RNAs, but mainly RNA [29]. MicroRNAs (miRNAs) are a class of non-coding small RNAs with less than 30 nucleotides in length, playing an important role in mediating cross-kingdom communication [62]. Moreover, studies revealed that these miRNAs, as key gene regulators, have the potential to regulate the target genes, thus exerting therapeutic effects [63]. For instance, miR159 from Prunus mume-derived VLNs has been found to significantly alleviate colitis in mice via selectively reducing the NEK7-NLRP3 interaction to block the assembly of inflammasomes and ultimately inhibit the activation of NLRP3 inflammasomes in macrophages [64]. In another study, Liu et al. [65] demonstrated that Pab-miR-396a-5p within Perilla frutescens leaf-derived VLNs, serving as a functional component, targeted the 3′-untranslated regions of plant heat shock protein 83a, leading to the suppression of nuclear factor-kappa B and Janus kinase/signal transducers and activators of transcription signaling, hence inhibiting the interleukin-17 signaling pathway in psoriasis. Moreover, miRNAs packaged in *Houttuynia cordata*-derived VLNs mediated cross-species regulation between respiratory RNA viruses and host-specific genes to exert antiviral effects [66].

It has been shown that these miRNAs have potential anti-cancer effects by specifically targeting signal pathways [63]. For example, VLNs derived from Brucea javanica effectively deliver functional miRNAs to breast cancer cells, thus significantly inhibiting both growth and metastasis through modulation of the PI3K/Akt/mTOR signaling pathway and promotion of apoptosis via the ROS/caspase pathway [67]. MiR18a, present in grapefruit-derived VLNs, has been found to inhibit colon tumor growth by targeting β-catenin, which enhances angiogenesis, proliferation, and epithelial integrity. Additionally, it suppresses liver invasion by metastatic colon cancer cells through activation of the IFN-γ/Irf2 axis in M1 macrophages, leading to IL-12-mediated recruitment and activation of NK and NKT cells [68,69]. Moreover, Wang et al. [70] found that miR167a in VLNs from broccoli may induce apoptosis in human pancreatic cancer cells by activating the PI3K-AKT signaling pathway and upregulating genes associated with pro-apoptotic effects.

#### 2.3.4. Unique Metabolite of Certain PDVLNs

During the biogenesis, PDVLNs preserve unique active metabolites from their source plants and retain similar therapeutic effects. Woith et al. held that these metabolites in PDVLNs were more likely to be associated with the lipophilicity of vesicle membrane structures rather than being actively packaged into EVs [71]. Various small-molecule active components have been reported, including polysaccharides, glucose, fructose, sucrose, alkaloids, glucosinolates, flavonoids, and others. These active metabolites possess numerous biological actions, such as anti-inflammatory, tissue repair, anti-aging, and anti-cancer capabilities [72,73]. For instance, Naringin, encapsulated in Rhizoma Drynariae-derived nanovesicles, demonstrated targeted affinity for bone tissue by promoting the proliferation and differentiation of osteoblasts via targeting ERα signaling, thereby ameliorating osteoporosis [74]. The same research team confirmed that monotropein was one of the main components of Morinda Officinalis-derived VLNs and was involved in the anti-osteoporosis effect of these VLNs [75].

The anti-tumor effects of these metabolites have gradually been revealed. For instance, a high concentration of 6-gingerol and 6-shogaol in GVLNs was detected by high-performance liquid chromatography with mass-spectrometry, and played a crucial therapeutic role in the anti-inflammatory and anti-cancer effects of GVLNs [76]. Wang et al. [77] further revealed that most of the shogaols are encapsulated in GVLNs using thin-layer chromatography rather than ginger extracts. In addition, lipidomic analysis of GVLNs indicated the presence of gingerols and shogaols and exhibited a remarkable inhibitory effect on tumor cells. Additionally, Chen et al. [78] suggested that cucumber sarcocarp-derived VLNs containing cucurbitacin B (CuB) possessed greater anti-cancer activity in non-small-cell lung cancer compared with free CuB, and speculated that it might be associated with improved bioavailability of CuB. Table 2 summarizes the tissue pretreatment, extraction strategies, and general physicochemical characterization of PDVLNs according to the latest research.

In summary, PDVLNs are rich in biological molecules such as lipids, proteins, and nucleic acids, as well as secondary metabolites. These components are necessary for mediating intercellular cross-border communication and material transfer, thus exerting a synergistic effect on human diseases, especially cancer [79]. Despite many lipidomics and proteomics studies conducted, PDVLNs protein markers have not yet been identified. Therefore, more in-depth studies are needed to identify their functional molecules, thus laying the foundation for their application in clinical nanomedicine.

**Table 2:** Extraction methods, shape, yields, and physical-chemical characterization of PDVLNs.

Plant	Part	Pretreatment	Extraction	Physical Property				Chemical Components	Refs.
				Morphology	Particle Size	Concentration or Yield	Surface Charge		
*Dioscorea japonica*	Root	Juicing	DUC + DGC	Round	168 nm and 328 nm	15 × 10^9^ particles/mL	-	RNA, proteins, and lipids	[80]
*Taraxacum mongolicum* Hand.-Mazz.	Whole plant	Squeezing	DUC + UF	Sphere-shaped	187 nm	5.75 × 10^11^ particles/mL	-	Proteins, lipids, and RNA	[81]
*Rhizoma Drynariae*	Root	Blending	DUC	Cup-shaped	75.7 ± 15.8 nm	4.17 × 10^11^ particles/mg	−43.2 ± 0.04 mV	RNAs, proteins, lipids, naringin, narirutin, and naringenin	[74,82]
*Pueraria lobata*	Root	Soaking	DUC	Round	119 to 163 nm	6 × 10^9^ particles/mL	-	-	[83]
*Pueraria lobata*	Root	Grinding	DUC + UF	Round	40 to 150 nm	1.0 × 10^11^ particles/mL		-	[84]
*Morinda Officinalis*	Root	Juicing	DUC	Round- or cup-shaped	61.24 ± 12.74 nm	2.25 × 10^12^ particles/mL	-	Nucleic acids, proteins, and lipids	[75]
*Rehmanniae Radix*	Root	Juicing	DUC + DGC	-	118 nm	2.6 × 10^6^ particles/mg	-	miRNAs	[82]
*Atractylodes macrocephala*	Root	Juicing	DUC	Round- or cup-shaped	90.61 ± 19.66 nm	6.27 × 10^11^ particles/mL	-	Nucleic acids, lipids, and metabolites	[85]
*Coptis chinensis*	Root	Juicing	DUC	Cup-shaped	55 to 120 nm	8.45 × 10^10^ particles/mL	-	-	[86]
*Platycodon grandiflorum*	Root	Juicing	DUC + DGC	Circular or elliptical	90 nm	1.4 × 10^8^ particles/mL	−23.5 mV	Proteins	[87]
*Lycium barbarum* L.	Fruit	Juicing	DUC + DGC	Round	171.0 nm	9.48 × 10^10^ ± 1.86 × 10^9^ particles/mL	-	Lipids, terpenoids, alkaloids, amino acids and derivatives, saccharides, and alcohol compounds	[88]
*Robinia pseudoacacia* L.	Flower	Grinding	Enzymolysis	Round	176.3 nm	4.92 × 10^14^ particles/kg	83.4 mV	Fatty acyls, glycerophospholipids, glycerolipids, sphingolipids, sterol lipids, prenol lipids, and polyketides	[89]
*Houttuynia cordata*	Leaf and flower	Juicing	DUC	Round	169.5 and 166.2 nm	6.67 × 10^10^ particles/g and 1.5 × 10^10^ particles/g	−23.04 ± 1.22 and −28.73 ± 0.87 mV	326 miRNAs	[57]
*Camellia sinensis*	Leaf	Juicing	Enzymolysis + DUC	Round	123.2 nm	1.16 × 10^10^ particles/g	−25 mV and −95 mV	Diacylglycerol, lysophosphatidyl choline, and N-arachidonylglycine	[90]
*Carthamus tinctorius* L.	Flower	Homogenating	DUC + DGC	Saucer- or cup-shaped	142.6 ± 0.7 nm	(11.49 ± 6.84) × 10^9^ particles/g	−22.64 ± 0.52 mV	CD63 and TSG101, miR166a-3p, miR159a, and miR170-5p	[91]
*Solanum lycopersicum*	Fruit	Juicing	DUC + DGC	Circular	120 to 140 nm	2.2 × 10^10^ particles/mL	−20 to −30 mV	-	[92]
*Aloe vera* L.	Peel	Soaking	DUC	Round shaped	145 ± 14 nm, 132 ±13 nm	6.5 ± 5.7 × 10^8^ particles/g	-	Carbohydrates and flavonoids	[93]

Note: DUC, Differential ultracentrifugation; DGC, density gradient centrifugation; UF, ultrafiltration.

## 3. Intrinsic Anti-Cancer Activity of PDVLNs

### 3.1. Administration Route and In Vivo Distribution in Tumor Model

The selection of an appropriate administration route is a critical prerequisite for ensuring the safety and therapeutic efficacy of PDVLNs. Previous studies have demonstrated that four administration routes have been employed in cancer treatment, including oral administration, intravenous administration, intraperitoneal administration, and intranasal administration. And the administration routes of PDVLNs are one of the most significant factors affecting their biodistribution. Oral administration represents the most patient-friendly strategy for PDVLN delivery, given that the safety profile of edible plants has been widely established through daily consumption [94]. Gao et al. [55] employed DiR as a labeling agent for MLVLNs to track their biodistribution within an orthotopic liver cancer mouse model. Strikingly, they observed that the fluorescence signals of MLVLNs were perfectly colocalized with liver tumors, demonstrating their inherent liver tumor-targeting capability. In addition, the findings indicated oral MLVLNs is a relatively safe administration, while intravenous administration of MLVLNs has the potential hepatic toxicity. In another report, Yang et al. [95] found thatboth intraperitoneal and oral *Platycodon grandiflorum*-derived VLNs (PGVLNs) had a favorable biosafety profile, but the biodistribution varied between the oral and intravenous administration routes. Compared with oral administration, intravenously administered nanoparticles demonstrated prolonged retention of fluorescence intensity in both the abdominal region and the brain, persisting for up to 48 hours. However, PGVLNs were retained within the gastrointestinal tract for a longer time when taken orally. Therefore, for PGVLNs, intravenous administration may be a more appropriate administration method, and PGVLNs may be able to penetrate the blood–brain barrier (BBB) and targeted brain regions owing to the obvious fluorescence signal detected in the brain regions. Different modes of administration can cause PDVLNs to exhibit very different biodistributions. Ou et al. [57] gave mice injections in the tail vein, intraperitoneal administration, and oral administration of vesicle-like nanovesicles from Catharanthus roseus (CEVLNs). It was found that CELNVs could be retained in the gastrointestinal tract for a long time after oral administration. In addition, CELNVs via intraperitoneal administration are mainly distributed in the immune organs, such as the thymus, spleen, pulmonary vasculature, liver, and kidney, while CELNVs injected intravenously into the bloodstream may face interception and rapid clearance by the liver and spleen. Moreover, when utilizing PDVLNs as drug delivery platforms for neurological therapeutics, intranasal administration offers a better method to deliver therapeutic agents to the brain by penetrating the BBB and providing precise brain-targeted drug transport. For instance, intranasal administration of grapefruit-derived VLNs coated with folic acid and polyethylenimine was selectively taken up by GL26 tumor cells and delivered miR-17 to GL26 brain tumors, thus retarding tumor growth in vivo [69]. Moreover, Mi et al. revealed that intranasal administration of Ganoderma lucidum-derived VLNs could effectively ameliorate cognitive disorder by decreasing inflammation in an alzheimer mouse model [96]. The finding indicates that intranasal delivery of PDVLNs provides a noninvasive therapeutic strategy for brain diseases.

The selection of administration routes should be tailored to target organs or cells, as these strategies can modulate the in vivo biodistribution of PDVLNs to a certain degree. Before their clinical translation and routine application, it is essential to elucidate the internalization mechanisms and biodistribution characteristics of PDVLNs. Further investigations are warranted to evaluate the bioavailability, tissue distribution, and practical therapeutic potential of PDVLNs in humans via diverse administration pathways.

### 3.2. The Therapeutic Mechanism in Different Cancers

#### 3.2.1. Modulating Oxidative Stress to Induce RCD

Oxidative stress, characterized by the imbalance between reactive oxygen species (ROS) production and antioxidant defense capacity, plays a dual role in tumor progression. Low levels of ROS promote cell proliferation and metastasis, while excessive ROS induce oxidative damage to DNA, proteins, and lipids, ultimately triggering tumor cell death [97,98]. RCD, also called programmed cell death, refers to the form of cell death that can be strictly regulated by a variety of biomacromolecules and genetic programs. Based on different regulatory mechanisms, RCD can be divided into three major subroutines, including autophagy, apoptosis, and other types of RCD (e.g., ferroptosis, pyroptosis, and anoikis) [99]. Recently, numerous studies have demonstrated that PDVLNs loaded with diverse active ingredients can regulate key oxidative stress signaling pathways in tumor cells, breaking their redox adaptation and triggering different types of RCD, such as apoptosis and ferroptosis. For example, tea flower-derived VLNs were found to promote the apoptosis of breast cancer cells by stimulating the intracellular ROS amplification process, which was attributed to the active chemical components within them, such as polyphenols and flavonoids [100]. Recent research has demonstrated that *Salvia chinensis Benth*-derived VLNs downregulate the nuclear translocation of Nrf2 by activating the AMPK pathway, suppressing the expression of downstream antioxidant genes and GSH synthesis, which leads to ROS overload and ferroptosis in hepatocellular carcinoma cells [101]. Similarly, Momordica charantia-derived VLNs have been shown to exert significant cytotoxicity against cervical cancer cells, which was mediated by Bcl-2/Bax/p-Akt-dependent apoptosis and ferroptosis by upregulating GPX4 [102].

#### 3.2.2. Arresting the Cell Cycle

PDVLNs can exert anti-cancer effects through the modulation of diverse signal pathways related to cell cycles. For instance, MLVLNs, rich in active small molecules (e.g., rutin and quercetin 3-O-glucoside), were reported to block cell arrest at the G0/G1 phase in hepatocellular carcinoma [55]. In addition, grapefruit-derived VLNs were found to trigger cell cycle arrest at the G2/M checkpoint, which was connected with the downregulation of cyclin B1 and B2 expression levels and upregulation of the cell cycle inhibitor P21 [68]. Lemon-derived VLNs inhibited the viability of gastric cancer cells and induced cell arrest at S-phase through the upregulation of DNA damage-45 alpha (GADD45α) gene [103]. Cucumber-derived VLNs have been proven to promote cell cycle arrest in G2/M phase by the suppression of STAT3 phosphorylation, which ultimately led to the proliferation inhibition of human non-small-cell lung cancer [78].

#### 3.2.3. Regulating Gut Microbiota

Maintaining a healthy and balanced gut microbiota plays a crucial role in protecting human health. Research has shown that disruption or deficiency of gut microbiota is closely related to the development and progression of tumors, such as metastasis and proliferation [16]. Apart from direct inhibition of tumor growth, PDVLNs as therapeutic agents can reshape gut microbiota homeostasis to exert anti-cancer effects [104]. For example, Chen et al. [100] found that VLNs from tea flowers could inhibit lung metastasis of breast cancer cells by microbiota modulation, including reducing the abundance of detrimental bacteria and increasing the percentage of beneficial bacteria [55]. Recent research by Teng et al. [105] revealed that GVLNs were preferentially taken up by gut bacteria and led to changes in the composition and localization of bacteria, as well as in host physiology. GVLNs were preferentially internalized by Lachnospiraceae and Lactobacillaceae, which was mediated by digalactosyldiacylglycerol and glycine, respectively. Further study indicated that alymi R159a-3p in GVLNs could improve programmed death-ligand 1 (PD-L1) antibody effect against melanoma by inhibiting the expression of recipient bacterial phospholipase C (PLC) and increasing the accumulation of gut product docosahexaenoic acid (DHA). Their findings provided a mechanistic insight into the impact of PDVLNs on the complex microbiota-tumor interaction.

#### 3.2.4. Reversing Immunosuppressive TME

TME is a dynamic ecosystem comprising diverse immune cell types, including fibroblasts, endothelial cells, pericytes, and various additional tissue-resident cell types [106]. The immunosuppressive TME, characterized by high levels of immunosuppressive cells (M2 macrophages, Tregs, MDSCs) and cytokines (IL-10, TGF-β), is a major barrier to cancer immunotherapy. PDVLNs have emerged as a potent immune modulator to rewire the TME towards an immunostimulatory state, thereby enhancing the immunotherapy. For instance, Cao et al. [56] found that GDNPs have been shown to repolarize the M2 to M1 phenotype both in vitro and in vivo via a TLR4-MyD88-dependent mechanism, which eventually inhibits tumor growth. Moreover, GDNPs could upregulate the secretion of chemokines CCL5 and CXCL9 for recruiting CD8^+^ T cells into the tumor bed, thereby significantly alleviating the immunosuppressive “cold” tumor phenotype and promoting immune checkpoint blockade therapy [107]. In another study, the team demonstrated that GDNPs reprogrammed macrophages to promote arginase-1 secretion by modulating the mTOR–T-bet axis, thereby alleviating T cell exhaustion [108].

**Figure 3:**
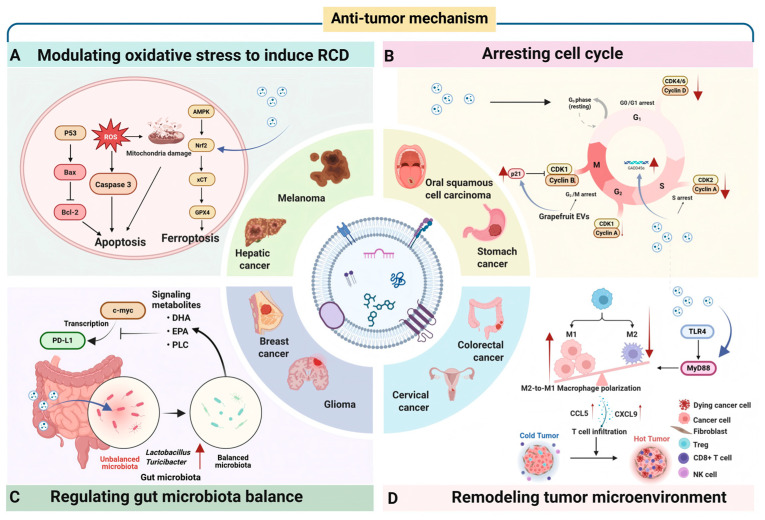
Illustration of the anti-tumor mechanisms of PDVLNs in cancer therapy. (**A**) Regulated cell death: PDVLNs induce cytotoxic effects by mediating regulated cell death (e.g., apoptosis by the activation of the caspase pathway); (**B**) Cell cycle arrest: PDVLNs inhibit tumor proliferation by inducing G1/G0, G1/S, or G2/M phase arrest; (**C**) Gut microbiota balance: PDVLNs regulate the proportion of gut microbiota or mediate related metabolites (e.g., DHA, EPA, and PLC); (**D**) Tumor immune microenvironment: PDVLNs alter macrophage polarization state or alleviate T-cell exhaustion to change cold tumor into hot tumor. DHA, docosahexaenoic acid; EPA, eicosapentaenoic acid; PLC, bacterial phospholipase C. Red arrows indicate upregulation or downregulation, while black arrows serve as indicators.

PDVLNs exert an inhibitory effect on cancer cell proliferation by modulating oxidative stress, cell cycle progression, gut microbiota, and the TME, as shown in Figure 3. These VLNs offer advantages such as scalable production capacity and a favorable safety profile, positioning them as promising candidates for future clinical studies. However, despite extensive research into the mechanisms underlying PDVLN-mediated effects, the specific bioactive components responsible for these biological activities remain to be fully identified. This knowledge gap highlights the need for further in-depth investigation. Table 3 illustrates the anti-tumor mechanisms of PDVLNs and the associated signal pathways in various cancers.

**Table 3:** The anti-tumor effects, potential mechanisms, and major ingredients of PDVLNs.

Plant Source	Cancer	Mechanism and Pathway of Action	Active Ingredient	Refs.
*Allium sativum* L.	A498 and A549	(i) Inhibit cancer cell proliferation (ii) Induce caspase-mediated apoptosis	-	[109]
*Citrus limon* L.	A549, SW480 and LAMA84 cells	(i) Induce TRAIL-mediated cell apoptosis	-	[110]
*Dendropanax morbifera*	B16BL6 mouse melanoma cell	(i) Reduce melanin content and tyrosinase, and tyrosinase-related proteins		[111]
*Camellia sinensis* [L.] O. Kuntze	Breast cancer	(i) ROS accumulation and microbiota modulation	-	[100]
*Camellia sinensis*	Breast cancer	(i) Pro-apoptosis via an increase in the accumulation of intracellular ROS and microbiota modulation	-	[112]
*Brassica oleracea* var. italica	Caco-2	(i) Toxic effects on cancer cells and apoptosis induction	ath-miR159a, ath-miR162a-3p, ath-miR166b-3p, and ath-miR396b-5p	[113]
*Momordica charantia* L.	Cervical cancer	(i) Activate the Bcl-2/Bax/p-Akt pathway (ii) Induce ferroptosis by upregulating GPX4	-	[102]
*Zingiber officinale* Roscoe	Colon cancer	(i) Decrease pro-inflammatory cytokines (ii) Trigger apoptosis	6-gingerol and 6-shogaol	[76]
*Zea mays* L.	Colon cancer	(i) Inhibit the proliferation of cancer cells (ii) Induce tumor necrosis factor-α release from RAW264.7 cells	-	[114]
*Citrus limon* L.	Gastric cancer	(i) Cause the gastric cancer cell cycle S-phase arrest (ii) Cell apoptosis	-	[103]
*Momordica charantia*	Glioma	(i) Inhibit glioma growth through PI3K/AKT pathway (ii) Prevent metastasis by reducing MMP9	-	[115]
*Asparagus cochinchinensis*	Hep G2, Hep 3B and SMMC-7721	(i) Increase vesicle circulation time and accumulation in tumor sites (i) Apoptosis-inducing effect	-	[15]
*Morus nigra* L.	Hepatocellular carcinoma	(i) Increase intracellular ROS and trigger mitochondrial damage (ii) Intestinal microbiota modulation	-	[55]
*Salvia chinensis* Benth	Hepatocellular carcinoma	(i) Induced ferroptosis by the AMPK/Nrf2/xCT axis	-	[101]
*Cannabis sativa* L.	Hepatocellular carcinoma	(i) Arrest the G0/G1 phase; induce cell death by activating mitochondrial-dependent apoptosis	D-9-tetrahydrocannabinol and cannabidiol	[116]
*Centella Asiatica*	HepG2	(i) Increase in ROS levels, mitochondrial damage (ii) Cell cycle arrest at the G1 phase (iii) Induce apoptosis	Rich miRNAs	[117]
*Boesenbergia rotunda* (L.) Mansf.	HT-29 and HCT116	(i) Induction of apoptosis	-	[118]
*Hedyotis diffusa* Willd.	Huh-7 cells	(i) Suppress the proliferation (ii) Induce apoptosis	miR-451, miR-486, miR-144, let-7, and miR-30	[119]
*Brassica oleracea* var. italica.	Human embryonic kidney 293 T cells	(i) Induce apoptosis in cancer cells by targeting IRS1	miR167a	[70]
*Artemisia annua*	Lung cancer	(i) Remold tumor-associated macrophages for tumor regression by cGAS-STING pathway	Mitochondrial DNA	[120]
*Zingiber officinale* Roscoe	Melanoma	(i) Induce cell cycle by mediating p53 signaling pathways (ii) Cause cancer cell apoptosis.	Gingerols and shogaols	[77]
*Zingiber officinale* Roscoe	Melanoma	(i) Elevate the DHA level to inhibit c-Myc-mediated transcription of PD-L1	AlymiR159a-3p	[105]
*Panax ginseng* C. A. Mey.	Melanoma	(i) Alter M2 polarized macrophages dependent on TLR4 and MyD88 signaling	-	[56]
*Panax ginseng* C. A. Mey.	Melanoma	(i) Promote T cell infiltration into tumors; alter “cold” tumor into “hot” tumor status	-	[107]
*Panax ginseng* C. A. Mey.	Melanoma	(i) Ameliorate T cell exhaustion by reprogramming macrophages via the mTOR-T-bet axis,	-	[108]
*Citrus paradisi* Macfad.	Melanoma	(i) Trigger cell cycle arrest at G2/M (ii) Upregulate cell cycle inhibitor p21	-	[68]
*Cucumis sativus* L.	Non-small-cell lung cancer	(i) Inhibit the vitality of tumor cells by suppressing STAT3 phosphorylation (ii) Induce cell cycle arrest in G2/M phase	Cucurbitacin B	[78]
*Momordica charantia* L.	Oral squamous cell carcinoma	(i) Induce S phase cell cycle arrest and apoptosis (ii) Reduce the drug resistance of cancer cells to chemotherapeutic agents	-	[121]
*Fallopia multiflora* (Thunb.) Haraldson	SMMC-7721	(i) Modulate signal pathways associated with the cell cycle	-	[122]
*Platycodon grandiflorum*	Triple-negative breast cancer	(i) Facilitate ROS and the polarization of tumor-associated macrophages toward the M1 phenotype (ii) Modulate the gut microbiota	-	[123]
*Brucea javanica*	Triple-negative breast cancer	(i) Retard tumor growth and metastasis via PI3K/Akt/mTOR pathway (ii) Promote ROS/caspase-mediated apoptosis	Ten active miRNAs	[67]

## 4. PDVLNs as Drug Delivery Carriers

**Figure 4:**
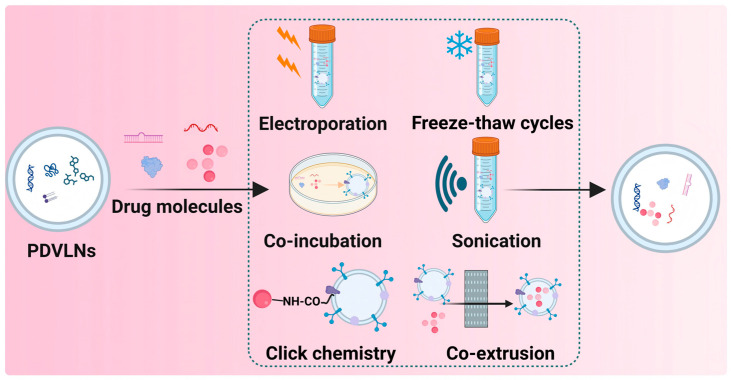
Drug loading strategies for PDVLNs.

Compared to synthetic nanoparticles, PDVLNs offer several advantages as drug delivery carriers, including the ability to achieve high biocompatibility, natural targeting capabilities, and high stability [124]. Leveraging their amphiphile, PDVLNs can encapsulate hydrophilic drugs in the cavity and allow hydrophobic drugs to load into the lipid bilayer. While PDVLNs serve as effective drug delivery carriers, the selection of an appropriate loading method is critical to maintain drug integrity and ensure high delivery efficiency [125,126]. Effective drug-loading strategies have been established for the loading of drugs with PDVLNs, such as electroporation, co-incubation, sonication, freeze–thaw cycles, and osmotic shock [127]. The characteristics of each loading method for drug delivery are summarized in Table 4 and Figure 4.

### 4.1. Drug Loading Methods

Electroporation has been widely employed for drug loading into MDVs [128]. By applying brief high-voltage pulses to generate an external electric field, electroporation induces the formation of numerous small hydrophilic pores on the PDVLNs membrane, allowing he desired cargo, such as DNA, RNA, and drugs, to enter PDVLNs, followed by rapid membrane resealing [129,130]. A key advantage of this approach lies in the operational simplicity and high loading efficiency.

#### 4.1.1. Electroporation

Co-incubation is the most prevalent strategy for passively loading drugs into PDVLNs. The drug and PDVLNs are incubated under optimized conditions, enabling the drug encapsulation within PDVLNs through diffusion and electrostatic interactions. This method is suitable for hydrophobic medicines [131].

#### 4.1.2. Sonication

Sonication of PDVLNs with target therapeutic molecules at a specific frequency induces membrane deformation via cavitation, mechanical, and thermal effects, thus facilitating the diffusion of cargo into the vesicle lumen. In certain cases, drugs may additionally adsorb to the outer membrane of PDVLNs. Notably, this approach offers enhanced loading efficiency, particularly for small RNAs; however, it is critical to employ moderate frequency parameters to mitigate excessive PDVLN membrane damage [132].

#### 4.1.3. Other Methods

Apart from the methods above, a variety of advanced delivery technologies, including freeze-thaw cycles, co-extrusion, and click chemistry, have been established as effective loading strategies. Notably, the freeze-thaw method, widely employed to facilitate liposome membrane fusion, allows for the efficient encapsulation of therapeutic agents into PDVLNs through repeated freeze-thaw cycles [133,134,135]. In comparison with conventional cross-linking approaches, click chemistry demonstrates superior efficiency in covalently conjugating drug molecules to the surface of PDVLNs, while preserving both the structural integrity and biological functionality of the vesicles [136].

### 4.2. PDVLNs as Natural Delivery Vehicles for Anti-Tumor Drugs

Anti-tumor drugs exhibit limited targeting specificity and are unable to selectively act on tumor cells. While effectively eliminating cancer cells, these agents also damage healthy cells and tissues, leading to adverse effects. This lack of selectivity represents a major challenge in current cancer therapy. Ensuring the stability of drugs and effective targeted delivery to a specific site are essential prerequisites for PDVLNs as drug delivery carriers [3]. Given the potential advantages of high biocompatibility, weak immune response after entering the body, and intrinsic targeted ability, PDVLNs have proven effective carriers for delivering anti-cancer agents, including chemotherapeutic drugs and nucleic acids [137]. Notably, in the TME, PDVLNs are attributed to specific molecular markers on their surface, enabling them to identify and bind the corresponding receptors on the tumor cell surface, thus achieving precision drug delivery. Wang et al. [138] demonstrated that grapefruit VLNs exhibit high performance in the co-delivery of folic acid and the chemotherapeutic drug paclitaxel (PTX) to colon tumor sites. In vivo experimental results showed that the treatment not only enhanced the therapeutic effect of PTX by targeting tumor tissues and inhibiting tumor growth, but also improved siRNA delivery to tumors. Huang et al. [139] found that edible and cationic-free kiwifruit-derived vesicles could mediate EGFR-targeted siRNA delivery, thereby inhibiting multidrug-resistant lung cancer. Additionally, cabbage-derived VLNs successfully transferred doxorubicin (DOX) to SW480 cells without compromising EV integrity or the anticancer efficacy of DOX [36].

PDVLNs exhibit promising potential as emerging nanocarriers in cancer therapy. With the ongoing advancement of related research and technological innovations, these developments are anticipated to enable more effective and safer therapeutic strategies for cancer treatment.

**Table 4:** The main advantages and disadvantages of loading strategies for PDVLNs.

Loading Strategy	Drug Loading Type	Merit	Limitation	Refs.
Electroporation	Hydrophilic drug, such as nucleic acid	(i) High efficiency (ii) Simple operation	(i) Easy to cause the accumulation and agglomeration	[140,141]
Co-incubation	Small-molecule hydrophobic drug molecules	(i) Simple operation (ii) Mild drug loading process	(i) External force is needed	[142]
Sonication	Hydrophilic drug	(i) High loading efficiency (ii) Uniform particle size	(i) Disrupt the structure of PDVLNs	[143]
Freezing and thawing method	Chemical compounds	(i) High drug loading efficiency	(i) Time-consuming (ii) Lead to protein inactivation (iii) PDVLNs aggregation	[128]
Osmotic shock method	Hydrophilic drugs	(i) High drug loading efficiency (ii) Easy operation (iii) Maintain the activity of PDVLNs	(i) The structure of the PDVLNs may be damaged (ii) Suitable to load only one drug	[134]
Co-extrusion	Fat-soluble and hydrophilic drugs	(i) High drug loading efficiency (ii) Simple operation (iii) Uniform particle size	(i) The integrity of PDVLNs is easily damaged	[143,144]
Click chemistry	Chemical compounds	(i) High efficiency	(i) The drug loading process is impacted by the environment	[135]

### 4.3. Engineering Strategies of PDVLNs for Cancer Therapy

As natural drug delivery platforms, PDVLNs possess inherent advantages but have certain drawbacks, such as low targeting abilities, rapid clearance, and poor uniformity. To address these challenges, a range of engineering modification technologies is needed to augment accurate drug delivery and enhance effective accumulation at the treatment site. In this section, we will explore the application of the targeted modification strategy of PDVLNs-based cancer therapy (Table 5).

#### 4.3.1. Surface Modification

Surface modification of PDVLNs is achieved by chemically conjugating specific targeting ligands such as peptides, antibodies, or aptamers onto the PDVLN surface. Modified PDVLNs can selectively target the lesion site by binding to the receptors on the surface of the target cells [145]. Ligands targeting cancer cell surface markers can direct PDVLNs more precisely to neoplastic tissues, thus reducing off-target effects and increasing the payload capacity of drugs.

Niu et al. [146] designed a novel drug delivery system where functional heparin was surface-modified onto grapefruit-derived VLNs loaded with DOX by chemical conjugation. The system could effectively promote the penetration of drugs into the BBB and enhance anti-glioma efficacy. In addition, grapefruit-derived VLNs after chemical modification with folic acid can also encapsulate and effectively deliver paclitaxel to colon tumor sites and enhance the therapeutic effect of paclitaxel in vivo experiment, but do not cross the placental barrier to cause cytotoxicity after intravenous administration (i.v.) [138]. Similarly, lemon-derived VLNs marked with heparin-cRGD-VLNs-DOX on the surface were found to overcome DOX-resistant ovarian cancer [147]. GVLNs modified with folic acid-displaying arrow-tail RNA nanoparticles on the surface precisely delivered siRNA to human oral epidermoid carcinoma cells and suppressed tumor growth [148]. This targeted delivery strategy significantly enhances the efficiency of drug absorption and therapeutic impact [149]. In addition to exogenous drugs, PDVLNs can also carry exogenous small RNAs. For example, grapefruit-derived VLNs after surface modification delivered exogenous miR17 and miR-18a to tumor cells for the inhibition of brain tumor progression and liver metastasis of colon cancer, respectively [69,150].

#### 4.3.2. Membrane Hybridization

Membrane hybridization modification technology is another promising strategy. The lipid bilayer membrane of PDVLNs can spontaneously be merged or coated with different cell types, such as immune cells or cancer cells, through physical techniques of ultrasonication, co-extrusion, and freeze-thaw cycles [151]. This hybridization strategy capitalizes on the complementary advantages of PDVLNs and more types of membranes, thereby exerting a synergistic effect on the regulation of the tumor immune microenvironment. For example, Huang et al. [152] fused the grapefruit-derived VLNs with the cell membranes of gingiva mesenchymal stem cells (GMSC) to prepare the MDV-plant hybrid nanovesicles. This hybrid membrane technology preserved the immunomodulatory capabilities of both grapefruit-derived VLNs and GMSC membranes. Sun et al. [153] developed bacteria-plant hybrid nanovesicles via the fusion of spinach leaf-derived thylakoid membranes with bacterial cell membranes. This hybrid membrane strategy exerts a synergistic effect on the eradication of primary solid tumors while inhibiting cancer metastasis.

Co-extrusion is the most commonly used preparation method, which is simple to operate and can be achieved by extruding a press. The membrane fusion technique can realize the biological orthogonal targeting of plant vesicles in vivo. Beyond enhancing the in vivo targeting capability and safety profile of PDVLNs, membrane fusion strategies enable the engineering of personalized cancer vaccines that confer protection against tumor recurrence and metastasis. For instance, Wang et al. [154] successfully developed a membrane-fused cancer vaccine via co-extrusion of GDNPs and autologous tumor cell membranes isolated from resected tumors. This PDVLN-based personalized tumor vaccine not only exhibits favorable biocompatibility but also effectively mitigates the risk of postoperative tumor recurrence and metastasis. Collectively, drug targeting, delivery, and drug resistance alleviation are two major developmental directions for engineered PDVLN application in cancer treatment.

#### 4.3.3. Stimuli-Responsive PDVLNs

Stimuli-responsive PDVLNs release drugs in response to specific TME signals (e.g., low pH, high glutathione, overexpressed enzymes), improving drug release precision and reducing side effects [18]. For example, pH-sensitive PDVLNs are stable in the neutral bloodstream but rapidly release drugs in the acidic TME. Stimuli-responsive modification further enhances the controllability and efficacy of PDVLN-based drug delivery systems. According to the type of stimuli, they can be divided into two major categories: endogenous stimuli-responsive PDVLNs (responding to TME or pathological site characteristics) and exogenous stimuli-responsive PDVLNs (responding to external artificial signals).

Endogenous stimuli are inherent physiological or pathological signals in vivo, such as pH, glutathione (GSH), ROS, and overexpressed enzymes. This type of PDVLN does not require external intervention, enabling autonomous and precise drug release at the target site, which is highly compatible with the concept of precision medicine. For example, Lu et al. [155] prepared an innovative ROS response system by incorporating dibenzocyclooctyne (DBCO)-NHS onto the surface of *Exocarpium Citri grandis*-derived VLNs. In the ROS-enriched environment, ROS-N_3_ will be hydrolyzed. The N_3_-DBCO facilitates the targeted accumulation of Exocarpium Citri grandis-derived VLNs at the target part through a “click” chemistry reaction. This research offers a valuable approach to developing multifunctional PDVLNs.

**Figure 5:**
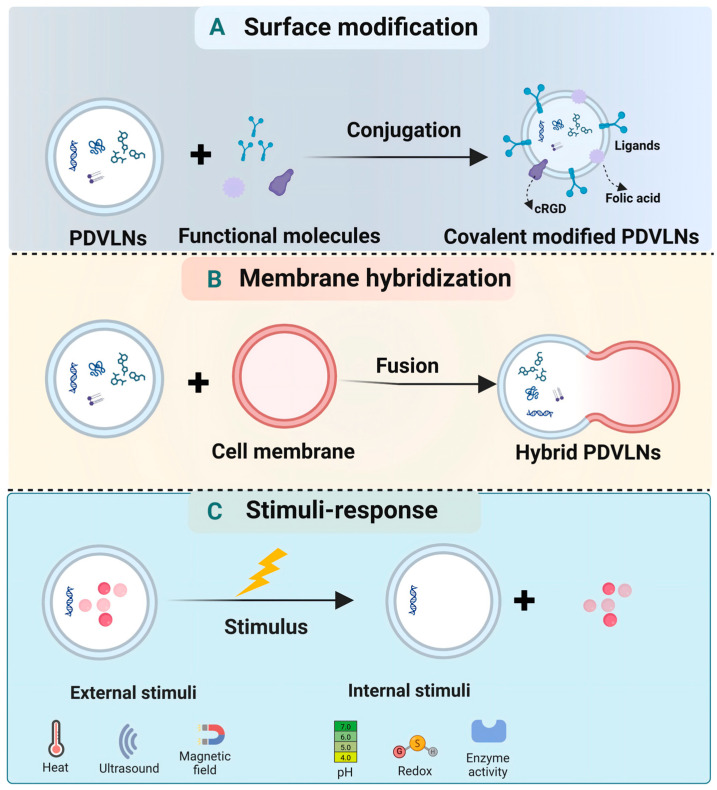
Engineering strategies to functionalize PDVLNs. (**A**) Surface modification. (**B**) Membrane hybridization. (**C**) Stimuli-responsive design.

Exogenous stimuli refer to artificially controlled external signals, such as light, ultrasound, temperature, and magnetic fields. Incorporating photosensitizers, thermosensitizers, sonosensitizers, or other stimulus-responsive materials into PDVLNs makes it possible to achieve targeted drug delivery [156]. The delivery system of PDVLN allows for on-demand and precise regulation of drug release time and location by controlling the intensity and duration of external stimuli, which is particularly suitable for the treatment of malignant tumors that require real-time monitoring and adjustment. Figure 5 illustrates the commonly employed engineering techniques and drug delivery strategies in PDVLNs. More preclinical investigations are required to accomplish clinical transformation in the future.

**Table 5:** Engineered PDVLNs for cancer therapy.

Plant Source	Loading Method	Modification Process	Cancer	Advantage	Refs.
Grapefruit	Co-incubation	Coated with the plasma membrane of leukocytes and deliver curcumin and Dox	Breast cancer and colon cancer	(i) Enhanced the homing ability to inflammatory tumor tissues	[69]
Grapefruit	Sonication	Coated with FA and polyethylenimine	Glioma	(i) Increase drug loading (ii) Targeting capacity, and alleviating the toxicity of the polyethylenimine	[150]
Aloe vera	Co-incubation	Deliver indocyanine green for phototherapy	Melanoma	(i) Promote the accumulation of drugs at the tumor site (ii) Enhance the anti-cancer efficacy	[73]
Ginger	Co-incubation	Coated with FA and siRNA	Human oral epidermoid carcinoma cells	(i) Increase the specific delivery of siRNA to tumors	[148]
Ginger	Sonication	Carry FA and Dox	Colon cancer	(i) Mediate targeted delivery of Dox to tumor cells (ii) Enhance the chemotherapeutic inhibition of tumor growth	[157]
Cabbage	Co-incubation	Deliver Dox and miR184	Colon cancer	(i) Improve the targeting effect of the drug	[42]
Spinach	Freeze-thaw cycle	Combine with outer membrane vesicles from *Escherichia coli* MG1655	Colon cancer	(i) Improve targeting accuracy (ii) Strengthen specific immune responses	[158]
Lemon	Co-extrusion	Fuse with 4T1 cancer cell membrane fragments	Breast cancer	(i) Target the homologous tumor	[159]

## 5. PDVLNs for Combination with Cancer Immunotherapy

**Figure 6:**
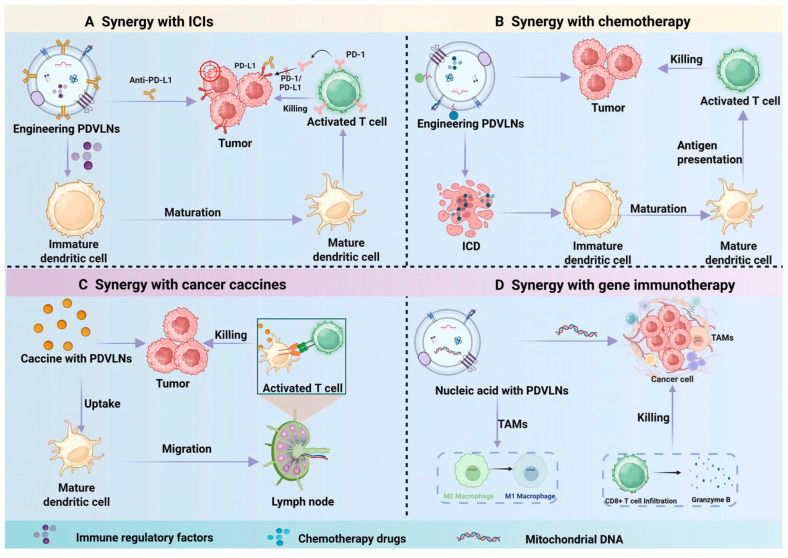
The synergistic applications of PDVLNs in combinational cancer immunotherapy. (**A**) Synergy with immune checkpoint inhibitors. (**B**) Synergy with chemotherapy. (**C**) Synergy with cancer vaccines. (**D**) Synergy with gene immunotherapy. TAMs, tumor-associated macrophages; ICD, immunogenic cell death.

Cancer immunotherapy stimulates the patient’s defenses to initiate an anti-cancer response [18]. Compared to traditional radiotherapy and chemotherapy, this treatment demonstrates improved safety profiles and a reduced incidence of adverse effects [160]. Chimeric antigen receptor T cell immunotherapy, immune checkpoint blocking, and cancer vaccines represent the three primary components of cancer immunotherapy [161]. Chemotherapy, phototherapy, hyperthermia, and gene therapy are additional anti-tumor modalities that can induce tumor immunogenic cell death, enhance T cell activation and dendritic cell (DC) maturation, and thereby elicit an immune response against the tumor. Research indicates PDVLNs not only act as standalone immunomodulatory agents but also can be engineered to composite particles to enhance immunotherapy, overcoming the limitations of single therapies, such as multidrug resistance in tumor cells and poor DC maturation [162]. When used in conjunction with cancer immunotherapy, engineered PDVLNs may exert a synergistic anti-tumor effect due to their superior anti-tumor efficacy (Table 6 and Figure 6).

### 5.1. Combination with Immune Checkpoint Inhibitors

Immune checkpoint inhibitors (ICIs) such as programmed death-1/programmed death-ligand 1 (PD-1/PD-L1) antibodies can restore anti-tumor immunity by reactivating exhausted effector T cells and have demonstrated promising clinical development in cancer treatment [163]. Unfortunately, their efficacy is often limited in “cold” tumors characterized by poor T-cell infiltration and an immunosuppressive TME [107]. In such contexts, DCs, which are essential for initiating adaptive immune responses, often exhibit impaired maturation and insufficient presentation of antigens, thereby further compromising effective T-cell priming [10]. Consequently, constructing integrated nano-delivery systems that directly activate T cells and enhance the ability of DCs to prime T cells effectively is crucial for boosting immunotherapy through combinatorial treatments. PDVLNs exhibit good biocompatibility, a nano-sized, and practical tissue penetration ability. They can be engineered to incorporate composite particles to achieve immunotherapy to inhibit tumor growth. For instance, Xu et al. [123] developed engineered nanocarriers using chemical modification to decorate the phospholipid bilayer of Glycyrrhiza uralensis Fisch roots-derived nanovesicles (GCNV) with PD-L1 antibodies. These nanocarriers demonstrated specific recognition of tumor cells and disrupted the PD-L1/PD-1 immunoinhibitory pathway to enhance T cell response. To further enhance DC maturation, they employed DMXAA (vadimezan), a STING agonist that activates the innate immune response by inducing type-I interferon production. Hence, the engineered GCNV system integrated a multifaceted therapeutic strategy that simultaneously inhibits tumor proliferation, activates T cells directly, and enhances DC activity, consequently exhibiting potent therapeutic efficacy against tumors. In addition, the latest research also demonstrated that garlic-derived VLNs could specifically migrate to the TME from the gut by increasing the level of chemokine CXCR3 in intestinal γδ T cells and remodel the TME and work synergistically with anti-PD-L1 to induce robust anti-tumor immunity [164].

### 5.2. Combination with Chemotherapy

Recent advances in tumor immunology have revealed that certain conventional chemotherapeutic agents can trigger anti-tumor immune responses and confer durable tumor suppression [165,166]. Notably, drugs such as DOX, oxaliplatin (OXA), and paclitaxel are capable of inducing immunogenic cell death (ICD), a form of RCD that activates the adaptive immune system to mount a potent and specific anti-tumor response [167,168]. When combined with chemotherapeutic agents, PDVLNs, owing to their intrinsic immunoregulatory activity, can serve as effective drug delivery vehicles or exert a synergistic therapeutic effect. For example, Guo et al. [169] employed DSPE-PEG2000 as a linker to functionalize turmeric-derived VLNs with the tumor-targeting ligand death receptor 5. These engineered VLNs were subsequently loaded with DOX via co-incubation. The obtained delivery system specifically targets glioma cells through targeted receptor-mediated recognition, inducing tumor cell death and enhancing natural killer (NK) cell activity by downregulating senescence-associated factors, thereby synergistically suppressing glioma progression. In another study, VLNs derived from citrus fruit were co-loaded with heparin sodium and a chimeric angiopep-2 (ANG) peptide. Under the tumor-localizing effect of heparin sodium, these VLNs efficiently delivered DOX to the tumor site. Within the EV cargo, bioactive compounds, including hesperidin, vitamin C, vitamin Eand the immune adjuvant ANG act in concert to potentiate anti-tumor immunity. Furthermore, DOX-induced ICD amplifies this immune activation. Together, this chemo-immunotherapeutic strategy demonstrates significantly superior tumor growth inhibition compared to monotherapy.

### 5.3. Combination with Cancer Vaccines

Cancer vaccines aim to induce specific anti-tumor immune responses by delivering tumor antigens, but their efficacy is limited by poor antigen delivery and weak immune activation. PDVLNs function as efficient antigen transport systems, protecting tumor antigens from degradation and delivering them to DCs. For example, Mu et al. [170] utilized GVLNs combined with antigen peptide OVA to construct a nanoscale cancer vaccine with dual targeting to DCs and lymph nodes (LNs), which contributed to triggering a strong adaptive response of ovalbumin (OVA)-specific CD8+ T cells. Notably, the cancer vaccine significantly enhanced the protective and therapeutic effects on the B16F10 melanoma model, with a complete remission rate of 40%. Likewise, Yang et al. utilized GVLNs and spinach-derived VLNs to construct functionality hydrogels, antigen peptide OVA was modified to the surface of spinach-derived VLNs, and oxidized sodium alginate was further modified to GVLNs by Schiff base reaction. The hydrogel can produce oxygen in the TME with a high level of hydrogen peroxide, reverse hypoxia-mediated immunosuppression, and promote the maturation of DCs and activation of T cells together with OVA peptides, triggering a strong anti-tumor immune response to inhibit the growth of the tumor [171].

### 5.4. Combination with Gene Immunotherapy

Cancer gene therapy encompasses strategies aimed at modulating or replacing dysregulated cancer-associated genes [172]. Current approaches predominantly utilize small interfering RNA (siRNA) or plasmid DNA to silence oncogenic targets in tumor cells [173]. To integrate gene therapy with immunotherapy, PDVLNs, particularly those exhibiting intrinsic immunomodulatory properties, are being harnessed as dual-functional platforms for the co-delivery of nucleic acid therapeutics and immune regulators. As efficient natural nanocarriers, PDVLNs can encapsulate and deliver siRNAs or plasmids to tumor sites, enabling simultaneous epigenetic reprogramming of cancer cells and modulation of immune responses. For instance, VLNs derived from *Brucea javanica* have been shown to deliver ten active miRNAs into 4T1 breast cancer cells, leading to ROS- and caspase-dependent apoptosis via suppression of the PI3K/Akt/mTOR signaling axis, while also inhibiting angiogenesis within the TME, thereby significantly suppressing 4T1 cell proliferation and metastasis [67]. Moreover, Liu et al. indicated that mitochondrial DNA (mtDNA) from *Artemisia annua*-derived VLNs could reshape the TME and promote repolarization of M2 macrophages toward the M1 phenotype via activating the cGAS-STING pathway in lung cancer and enhance CD8+ T infiltration [120]. These findings collectively underscore the potential of engineered PDVLNs as versatile co-delivery systems capable of synergistically combining gene silencing with immune activation for enhanced cancer therapy.

**Table 6:** The application of PDVLNs for combinational cancer immunotherapy.

Combination Therapy	Source	Application	Refs.
Immune checkpoint inhibitors	Wild Glycyrrhiza uralensis Fisch roots	(i) Co-deliver immune checkpoint inhibitors; (ii) Activate T cells and promote the maturation of dendritic cells	[123]
	Garlic	(i) Promoting immune checkpoint blockade therapy	[164]
Chemotherapy	Ginseng	(i) Co-deliver autologous tumor antigens and immune adjuvants; (ii) Contribute to immune regulation	[154]
	Citrus fruit	(i) Contain anti-tumor bioactive components; (ii) Load chemotherapy drugs and immune adjuvants; (iii) Combine chemotherapy and immunotherapy	[174]
	Turmeric	(i) Co-deliver chemotherapy drugs and antibodies; (ii) Combine chemotherapy and immunotherapy	[169]
	Ginger	(i) Enhance tumor targeting; (ii) Amplify drug sensitivity	[7]
Cancer vaccines	Ginseng	(i) Co-deliver autologous tumor antigens; (ii) Promote the maturation of dendritic cells	[170]
	Cannabis sativa Roots	(i) Promote the maturation of dendritic cells; (ii) Reinforce immune cell responses	[175]
	Ginseng and spinach	(i) Promote the maturation of dendritic cells and activation of T cells; (ii) Triggering a strong anti-tumor immune response	[171]
Gene immunotherapy	Artemisia annua	(i) Deliver autologous miRNA for cancer therapy	[120]
	Brucea javanica	(i) Deliver autologous mitochondrial DNA for cancer therapy	[67]

## 6. Clinical Transformation of PDVLNs

Several species of mammalian exosomes are currently undergoing clinical trials [176]. In contrast, the study on PDVLNs is relatively new, having only been around for ten years, but they have shown promising development prospects in both basic research and clinical transformation areas. At present, five clinical trials focusing on PDVLNs have been registered on Clinicaltrials.gov (NCT01668849, NCT04879810, NCT01294072, NCT03493984; NCT04698447) (Table 7). According to the latest registration information, grape-derived VLNs, also referred to as grape extract, have been employed to treat oral mucositis triggered by radiotherapy and chemotherapy for head and neck cancer and have entered Phase I in June 2022 (NCT01668849). In another trial investigation, leveraging their robust capacity to encapsulate hydrophobic drugs, grape-derived VLNs were utilized as carriers for curcumin delivery in colon cancer therapy (NCT01294072) [177]. This drug delivery system effectively overcomes the challenges associated with curcumin administration and enhances its stability and bioavailability. In addition, ginger and aloe-derived VLNs are applied to alleviate insulin resistance and chronic inflammation in patients diagnosed with polycystic ovary syndrome (NCT03493984). Unfortunately, the trial has been withdrawn due to failure to enroll participants. Currently, an ongoing clinical trial is assessing the efficacy of lemon-derived VLNs in modulating cardiometabolic risk factors in subjects with metabolic syndrome (NCT04698447). At present, current clinical studies indicate that PDVLNs-based therapies exhibit preliminary safety and efficacy profiles. However, substantial data from these trials remain inaccessible, likely due to ongoing studies, pending submissions of results, or the absence of mandatory data-sharing policies. Considering the paucity of clinical trials involving PDVLNs, it is imperative to facilitate additional clinical investigations to substantiate their therapeutic potential [178].

## 7. Challenges and Future Perspectives

Natural PDVLNs have emerged as potential candidates for cancer therapy owing to their inherent advantages in drug delivery and pharmacological activities, and are increasingly used for combined immunotherapy. The paper summarizes the latest advancements of PDVLNs, including the formation mechanism, the major components, the anti-tumor mechanism, and the application as anti-tumor delivery carriers, highlighting their synergistic effect with tumor immunotherapy. Nevertheless, despite the promising potential of PDVLNs in cancer treatment, there are several challenges and limitations that need to be addressed before their clinical translation.

The isolation and characterization of PDVLNs are not well standardized in different experiments, which directly contributes to their diversity and heterogeneity and impedes their commercialization. Notably, the use of fresh plants, particularly medicinal species, is frequently constrained by seasonality and preservation technologies [87], which in turn leads to variability in PDVLNs from the same plant source. In addition, PDVLNs exhibit significant variability across plant species, tissues (roots, leaves, fruits), and growth conditions (climate, soil, cultivation methods, and growth stage), which may result in inconsistent composition, bioactivity, and therapeutic effects. Even for PDVLNs from the same plant source, different characterization methods often yield inconsistent results. For instance, the mean diameters of MLVLNs measured by atomic force microscopy (AFM) and TEM (100 nm) were significantly smaller than that determined by dynamic light scattering (DLS, 162.1 nm) [55]. This size disparity is a well-recognized phenomenon, primarily arising from the intrinsic differences between the techniques. TEM/AFM measures the dry, collapsed size of PDVLNs under vacuum or solid-state conditions, while DLS quantifies the hydrodynamic diameter of hydrated vesicles in suspension, including the hydration shell and potential aggregates. Notably, this discrepancy can be mitigated by standardized sample preparation (e.g., consistent dilution ratio, avoidance of aggregation) and method calibration, as reported in previous studies [179]. Moreover, the potential presence of contaminants, such as pesticides and heavy metals, originating from source plant materials in isolated PDVLNs constitutes a critical safety concern that warrants rigorous assessment. However, the current research on this topic is still in its infancy, with few studies focusing on the types, concentrations, and toxicological effects of such contaminants. Future studies should establish standardized detection methods for PDVLN contaminants and optimize purification protocols (e.g., multiple washing steps, affinity chromatography) to reduce pollutant residues, ensuring the safety of PDVLN-based therapies. Unlike single chemical entities, PDVLNs are composed of multilocular assemblies, which not only advance their potential for treating various diseases but also add complexity to quality control during the preparation [123]. In mammalian systems, well-recognized biomarkers (e.g., CD9, CD63, CD81) are enriched in the membranes of exosomes and often used as exosome biomarkers, facilitating their clinical translation [180]. In contrast, the absence of quality control biomarkers, including proteins, nucleic acids, lipids, and unique metabolites, poses a formidable challenge to the standardized identification or verifying whether they meet the traditional definition of EVs. Although some proteins, such as HSP60, HSP70, HSP80, and HSP90, have been identified in PDVLN, they lack cross-species validation and cannot be used as universal standards for PDVLN identification. Lastly, most studies rarely provide detailed information on the specific harvest season or growth stage of their source plants. This ambiguity and lack of transparency represent a major cause of discrepancies among studies, especially PDVLNs output, even when derived from the same plant species. Hence, establishing a standardized evaluation system for PDVLNs requires the integrated consideration of multiple factors, such as detailed plant source, potential biomarkers, and specific extraction parameters. For practical treatment regimens, the balance between yield and purity must be considered. For instance, DUC combined with DGC can achieve moderate yield and high purity, reducing the required plant matter, which is more feasible for clinical translation. Future optimization of isolation protocols should focus on improving yield while maintaining purity, such as modifying centrifugation parameters or developing novel composite isolation techniques, to minimize the volume of plant material needed and promote the repeatability of PDVLN-based therapies. Multiple studies have demonstrated that PDVLNs can exert antitumor effects by regulating cell cycle, RCD, as well as gut microbiota and their metabolites, and TME, but many specific receptor interactions and signaling pathways remain incompletely understood. Moreover, the key material basis responsible for the anti-cancer efficacy of most PDVLNs has not yet been fully elucidated. The pharmacokinetics and biodistribution of PDVLNs remain inadequately characterized. Their in vivo absorption, distribution, metabolism, and excretion (ADME) profiles vary with plant sources and administration routes, and the lack of long-term tracking data hinders dose optimization. Notably, current in vivo cancer models (e.g., immunocompromised mice bearing xenografts) have inherent limitations. They fail to fully recapitulate the complexity of human tumor heterogeneity, immune microenvironment, and drug metabolism, leading to potential discrepancies between preclinical efficacy and clinical outcomes. In addition, the reproducibility and robustness of reported anti-cancer and immunomodulatory activities are compromised by non-standardized isolation protocols, lack of uniform quality control criteria, and insufficient multi-center validation, which may raise concerns about the reliability of PDVLN-based therapies.

Long-term safety and immunotoxicity concerns cannot be ignored. Although PDVLNs exhibit low immunogenicity in short-term studies, repeated administration may induce immune responses (e.g., antibody production) or cumulative toxicity (e.g., organ damage), which urgently requires systematic safety evaluation in long-term preclinical studies [181]. When cellular exposure to these foreign substances leads to potential toxicity and immune rejection reactions. According to a recent study, researchers expressed concerns about the potential risks of PDVLNs by intravenous administration. After high-dose intravenous injection of tea flower-derived VLNs in breast cancer, a strong immune response was triggered, and the indicators related to hepatorenal toxicity were significantly elevated, indicating their potential hepatorenal toxicity. However, there were no adverse reactions when these VLNs were taken orally at both low and high doses [100]. Hence, rigorous orthogonal animal studies and preclinical trials for systemic toxicity assessments of PDVLNs are essential before formal clinical practice. Research on PDVLNs as drug delivery vehicles remains relatively limited, and their therapeutic potential has yet to be fully elucidated. Despite their favorable biocompatibility as drug delivery platforms, PDVLNs face significant challenges regarding cost efficiency, drug-loading capacity, tissue targeting precision, and controlled release performance. Hence, novel drug delivery techniques must be developed to enhance the therapeutic efficacy and drug delivery efficiency of PDVLNs. In addition, the impact of the engineering intervention of PDVLNs on its active ingredients needs to be further investigated to guarantee that the engineering treatment does not alter its original properties. Finally, regulatory hurdles persist due to the lack of standardized production processes, quality control criteria, and clear classification of PDVLNs (e.g., as drugs, medical devices, or supplements), which delays approval procedures.

In future PDVLNs research, the production optimization strategies reported for artificial nanomaterials may offer valuable insights for addressing PDVLNs’ production challenges. For instance, scalable culture systems (e.g., bioreactors with controlled microenvironments), modulation of process parameters (pH, temperature, nutrient supply), and genetic engineering of source plants have been proven effective in exosome large-scale production [182]. These strategies could be adapted to PDVLNs to overcome current limitations such as low yield, batch-to-batch variability, and high production costs, laying a foundation for their industrialization and clinical translation. In order to further promote the discovery of PDVLNs biomarkers, combined transcriptomics, proteomics, lipidomics, and metabolomics can systematically profile the molecular landscape of PDVLNs from different sources, identify conserved molecular signatures across species, and screen potential core biomarkers. Moreover, the applications of artificial intelligence-assisted profiling in PDVLNs offer an efficient tool for processing large-scale multiomics data, eliminating the interference of plant species and isolation methods, and accurately identifying stable biomarkers. Furthermore, establishing a public database documenting the PDVLNs’ isolation (e.g., standardized centrifugation speed and time, unified buffer composition) and characterization (e.g., minimum requirements for particle size, zeta potential, and biomarker detection), and purity control (e.g., limit of impurity protein content) is of great significance for promoting the openness and transparency of PDVLNs research. To a certain extent, it can also facilitate the standardized isolation, characterization, and quality control of PDVLNs.

Multidisciplinary approaches should be adopted to facilitate an in-depth understanding of the biological mechanisms of PDVLNs and foster innovative immunotherapy platforms. For example, the implementation of an integrated multi-omics approach, encompassing transcriptomics, proteomics, and metabolomics, is necessary to systematically delineate the downstream therapeutic effects and identify regulatory targets of signaling pathways involved in cancer immunotherapy. Additionally, single-cell sequencing technologies provide high-resolution tools for in-depth analysis of immune cell heterogeneity and interaction mechanisms mediated by PDVLNs. Personalized PDVLN-based immunotherapies can be developed based on the genetic background, tumor type, and immune status of individual patients. For example, personalized PDVLN vaccines loaded with patient-specific neoantigens can achieve precise anti-tumor immunity. The combination of PDVLNs with other emerging technologies (e.g., gene editing, bispecific antibodies) will further expand their application in personalized cancer immunotherapy.

The clinical translation of PDVLNs requires close cooperation between multiple disciplines, including nanomedicine, cancer immunology, botany, pharmacology, and clinical medicine. The development of GMP-compliant production processes for PDVLNs is essential for meeting clinical requirements. Additionally, well-designed preclinical studies (e.g., large animal models) and clinical trials (e.g., phase I/II clinical trials to evaluate safety and preliminary efficacy) are needed to promote the clinical application of PDVLNs. The establishment of international cooperation platforms can facilitate the sharing of resources and technologies, accelerating the development of PDVLN-based cancer immunotherapies.

## 8. Conclusions

Overall, research on PDVLNs as natural nanomaterials for cancer therapy and their synergistic application with cancer immunotherapy is advancing rapidly and achieving remarkable progress. (i) PDVLNs possess diverse biogenesis pathways and rich active components, which underpin their intrinsic anti-tumor activities. (ii) As natural drug delivery carriers, PDVLNs can encapsulate various anti-tumor agents via multiple loading strategies, and engineering modifications further enhance their targeting ability and delivery efficiency. (iii) PDVLNs exert synergistic anti-tumor effects when combined with cancer immunotherapies by acting as both immunomodulators and co-delivery platforms.

Although the field continues to face significant challenges in isolation and characterization protocols, in vivo pharmacokinetics, and long-term safety data, the integration of multidisciplinary technologies coupled with ongoing enhancements to clinical translation frameworks positions PDVLNs as a promising next-generation immunomodulatory nanoplatform for cancer therapy. In summary, PDVLNs possess unique biological properties, including intrinsic biocompatibility, low immunogenicity, natural bioactivity, and versatile drug loading capacity, which make them a promising alternative to MDVs and synthetic nanocarriers in oncology research.

## Figures and Tables

**Table 7:** Summary of clinical trials involving PDVLNs for various medical conditions.

Plant Source	Applications	Phase	Recruitment Status	Cargo	Last Update Posted	NCT Number
Grape	Colon cancer	I	Recruiting	Curcumin	20 December 2023	NCT01294072
Grape	Head and neck cancer/oral mucositis	I	Completed	/	9 August 2022	NCT01668849
Ginger	Irritable bowel disease	Not applicable	Completed	Curcumin	3 November 2022	NCT04879810
Ginger and Aloe	Polycystic ovary syndrome	Not applicable	Withdrawn	/	16 March 2021	NCT03493984
Lemon	Healthy volunteers/metabolic syndrome	Not applicable	Unknown	/	/	NCT04698447

## Data Availability

No new data were created or analyzed in this study. Data sharing is not applicable to this article.

## Abbreviations

The following abbreviations are used in this manuscript:

MDVs	Mammalian-derived extracellular vesicles
TME	Tumor microenvironment
PDVLNs	Plant-derived vesicle-like nanoparticles
VLNs	Vesicle-like nanoparticles
EVs	Extracellular vesicles
RCD	Regulated cell death
MVBs	Multivesicular bodies
TEM	Transmission electron microscope
EXPO	Exocyst-positive organelle
LEs	Late endosomes
ILVs	Intraluminal vesicles
PM	Plasma membrane
UC	Ultracentrifugation
DGC	Density gradient centrifugation
UF	Ultrafiltration
UCG	Ultrafiltration centrifugation
SEC	Size exclusion chromatography
PEG	Golyethylene glycol
GVLNs	Ginger-derived vesicle-like nanoparticles
GDNPs	ginseng-derived nanoparticles
PA	Phosphatidic acid
MGDG	Monogalactosyldiacylglycerol
miRNAs	microRNAs
MLVLNs	*Morus nigra* L. leaves-derived vesicle-like nanoparticles
PD-L1	Programmed death-ligand 1
PD-1/PD-L1	Programmed death-1/programmed death-ligand 1
DOX	Doxorubicin
DCs	Dendritic cells
OXA	oxaliplatin
ICD	immunogenic cell death
